# LncRNA MSC-AS1 Is a Diagnostic Biomarker and Predicts Poor Prognosis in Patients With Gastric Cancer by Integrated Bioinformatics Analysis

**DOI:** 10.3389/fmed.2021.795427

**Published:** 2021-12-02

**Authors:** Wei Yang, Fusheng Ge, Shuaibing Lu, Zhiming Shan, Liangqun Peng, Junhui Chai, Hongxing Liu, Baodong Li, Zhandong Zhang, Jinxi Huang, Yawei Hua, Yonglei Zhang

**Affiliations:** ^1^Department of General Surgery, The Affiliated Tumor Hospital of Zhengzhou University, Henan Cancer Hospital, Zhengzhou, China; ^2^Clinical Laboratory, The Children's Hospital of Henan Province, Zhengzhou Children's Hospital, The Affiliated Children Hospital of Zhengzhou University, Zhengzhou, China

**Keywords:** lncRNA MSC-AS1, prognosis, gastric cancer, bioinformatics, biomarker

## Abstract

Numerous studies have shown that long uncoded RNA (lncRNA) MSC-AS1 may play an important role in the occurrence and development of some types of cancer. However, its role in gastric cancer has rarely been discussed. This study aimed to clarify the association between lncRNA MSC-AS1 and gastric cancer using The Cancer Genome Atlas (TCGA) database. We determined the expression of MSC-AS1 using the Wilcoxon rank sum test; in addition, logistic regression was applied to evaluate the association between MSC-AS1 and clinicopathological characteristics. Also, Kaplan-Meier and Cox regression were used to evaluate the relationship between MSC-AS1 and survival. A nomogram was conducted to predict the impact of MSC-AS1 on prognosis. Moreover, Gene Set enrichment analysis (GSEA) was performed to annotate the biological function of MSC-AS1. Quantitative analysis of immune infiltration was carried out by single-set GSEA (ssGSEA). The MSC-AS1 level was elevated in gastric cancer tissues. An increased MSC-AS1 level was significantly correlated with T stage (odds ratio [OR] = 2.55 for T3 and T4 vs. T1 and T2), histological type (OR = 5.28 for diffuse type vs. tubular type), histological grade (OR = 3.09 for grade 3 vs. grades 1 and 2), *TP53* status (OR = 0.55 for mutated vs. wild type), and *PIK*3CA status (OR = 0.55 for mutated vs. wild type) (all *p* < 0.05) by univariate logistic regression. Kaplan-Meier survival analysis showed high MSC-AS1 expression had a poor overall survival [hazard ratio (HR) = 1.75; 95% confidence interval (CI): 1.25–2.45; *p* = 0.001] and progression-free interval (HR = 1.47; 95% CI: 1.03–2.10; *p* = 0.034). Multivariate survival analysis revealed that MSC-AS1 expression (HR = 1.681; 95% CI: 1.057–2.673; *p* = 0.028) was independently correlated with overall survival. GSEA demonstrated that the P38/MAPK pathway, the VEGF pathway, the cell adhesion molecules cams, the NOD-like receptor signaling pathway were differentially enriched in the high MSC-AS1 expression phenotype. SsGSEA and Spearman correlation revealed the relationships between MSC-AS1 and macrophages, NK cells, and Tems were the strongest. Coregulatory proteins were included in the PPI network. Upregulated lncRNA MSC-AS1 might be a potential biomarker for the diagnosis and prognosis of gastric cancer.

## Introduction

Gastric cancer is one of the top five cancers and a leading cause of cancer-related deaths around the world, regardless of country development (1). The mortality of gastric cancer is high, and the prognosis is poor (2). Because of the diet characteristics in China, the lack of awareness about screening, and other reasons, the disease may have progressed to an advanced stage at the time of discovery (3). Gastric cancer is insidious, without specific symptoms, and difficult to recognize at an early stage (4). Currently, the diagnosis of gastric cancer is based mainly on gastroscopy and histological examination, but gastroscopy and pathological biopsy are invasive procedures. Because of associated pain, patients may be unwilling to undergo gastroscopy, and examination costs are high and rely on the doctor's operative ability. Previous chemotherapy-based treatments only extend the median overall survival time of patients with advanced gastric cancer by 7-11 months (4). The prognosis of patients with early gastric cancer is good, but the prognosis of patients with advanced gastric cancer is poor because of the lack of effective targeted drugs and the susceptibility to drug resistance. Currently, the only drugs approved for targeted therapy of advanced gastric cancer are trastuzumab, ramucirumab, apatinib, and papolizumab; the clinical application of these targeted drugs is challenging (5). The currently available biomarkers that predict prognosis have some limitations resulting from tumor heterogeneity; thus, the field needs new biomarkers as prognostic indicators to effectively enhance prognosis and individualized treatment. In recent years, the search for indicators that influence the development and prognosis of gastric cancer at the gene level and that guide the development of targeted therapy has become prevalent in the field of advanced gastric cancer. Serum or plasma tumor markers are substances synthesized directly by tumor cells or released into the blood by non-tumor cells—for example, cancer and tumor suppressor gene products, enzymes, isozymes, carcinoembryonic antigens, and tumor-related antigens. These substances are commonly used to detect gastric cancer and to predict the prognosis of patients with gastric cancer. However, no single marker with high sensitivity and specificity exists, and most available markers must be combined for detection and analysis to reduce the misdiagnosis rate.

With the rapid progress of whole-genome sequencing technology, approximately 2% of the genes in the genome have been found to have protein-coding functions. Approximately 90% of the remaining genes are non-coding genes (i.e., do not have the function of encoding proteins). The ubiquitous non-coding RNAs in the human body are microRNA (miRNA) and long non-coding RNA (lncRNA).

lncRNAs, which are 200 nucleotides in length, are a series of single-stranded RNA molecules that have no protein-coding functions (6). Studies have shown, though, that deregulated lncRNAs could participate in vital biological processes of various carcinomas, including gastric cancer. Many studies have confirmed that lncRNA can regulate cell proliferation, differentiation, apoptosis, invasion, and metastasis and that it is involved in the occurrence, development, and metastasis of gastric cancer. Therefore, it can be used as a diagnostic marker in the diagnosis and prognosis of gastric cancer. Recently, studies have proven that lncRNA can regulate the invasion and metastasis of gastric cancer cells through myriad mechanisms. Abnormal expression of lncRNA in gastric cancer tissues may influence cancer development; however, the mechanism behind lncRNA actions in gastric cancer is still unclear.

Using gastric cancer RNA-sequencing (RNA-seq) data from The Cancer Genome Atlas (TCGA), the differences in expression of lncRNA between tumor and normal samples of patients with gastric cancer were analyzed, and the correlation between the expression of lncRNA and the clinicopathological indicators was studied. By analyzing the prognosis of cancer in relation to the presence of lncRNA, a multivariate Cox regression model based on lncRNA and clinicopathological features was constructed. A nomogram was used to demonstrate the survival probability estimation method and to analyze its predictive efficiency. The samples were then grouped according to the expression of a single gene; high and low-expression groups were distinguished, and the differential expression of reverse transcriptome was analyzed. Enrichment analysis of Gene Ontology (GO)/Kyoto Encyclopedia of Genes and Genomes (KEGG)/Gene Set Enrichment Analysis (GSEA) was carried out for the different genes. Analysis revealed that the expression of a single gene is highly correlated with particular genes and their functional pathways. Finally, by analyzing the correlation between the expression of single gene and the immune infiltration, the possible mechanism between the expression of that single gene and the development of a tumor was explored.

To date, a novel lncRNA molecule, MSC-AS1, has been identified as a key regulator of tumor development (7–10). One study found that MSC-AS1 promotes the progression of liver cancer by increasing the expression of PGK1 (11), Another study showed that MSC-AS1 increased nasopharyngeal carcinoma by regulating Mir-524-5p/NR4A2 (12). However, its role in gastric cancer has rarely been discussed. This study aimed to clarify the association between lncRNA MSC-AS1 and gastric cancer using The Cancer Genome Atlas (TCGA) database. We found that the expression level of lncRNA musculin antisense RNA1 (MSC-AS1) in gastric cancer tissues was significantly higher than the level in para-carcinoma tissues. Elevated lncRNA MSC-AS1 was related to advanced clinicopathological features. Kaplan-Meier analysis showed that the 5-year progression-free survival and overall survival of patients with high lncRNA MSC-AS1 expression were significantly higher than those in patients with low lncRNA expression. These results suggest that lncRNA MSC-AS1 might be an independent biomarker of poor outcomes for stomach cancer.

## Materials and Methods

### Source and Processing of Bioinformatics Data

RNA-seq data and patient clinicopathological information from the gastric cancer project were downloaded from a publicly available cancer database, The Cancer Genome Atlas (TCGA), using R software; the deadline for data collection was August 26, 2020. Overall, 375 patients with both survival time data and lncRNA MSC-AS1 expression data were screened. Gender, race, age, histological type, residual tumor, histological grade, anatomic neoplasm subdivision, reflux history, antireflux treatment, Barrett's esophagus, *TP53* status, *PIK3CA* status, T stage (depth of invasion), N stage (lymph node metastasis), M stage, and TNM stage (according to the 8th edition of the American Joint Committee on Cancer's TNM cancer staging system) data were obtained for selected patients from the 375 tissue samples as the gastric cancer group; data for patients representing 32 para-cancer samples were taken as the normal group. Data for patients with overall survival shorter than 30 days were excluded. The format for downloading the data were the level-3 high-throughput expression profile-fragments per kilobase of transcript per million mapped reads (HTSeq-FPKM) data and HTSeq-counts. The level-3 HTSeq- FPKM data of RNA-seq were converted into transcripts per million reads (TPM) format for subsequent analysis. Individuals with data that were not available or for whom clinical information was unknown were considered missing values. The Wilcoxon rank sum test and the Wilcoxon signed-rank test were used to compare the expression levels of lncRNA MSC-AS1 in paired or unpaired tumor samples and in control samples, respectively. According to the level of single gene expression, the group of tumor samples was divided into high and low-expression groups (median as the cutoff value). This research fully complied with the public guidelines of TCGA.

### Differential Expression Analysis

After pre-processing the data, the qualified HTSeq-counts format data were obtained and divided into high and low-expression groups according to the expression of lncRNA MSC-AS1 in the tumor sample. Then, differentially expressed genes (DEGs) were obtained using the DESeq2 package (13). |Log2 fold change (FC)| > 1.5 and adjusted *p* < 0.01 were used as the screening threshold for the differentially expressed genes (DEGs) analysis.

### General Enrichment Analysis

For the differential lncRNAs obtained between single lncRNA high/low-expression groups, additional GO enrichment analysis was performed to clarify the biological processes, molecular functions, and cellular components involved in these lncRNAs. At the same time, KEGG signaling pathway analysis was conducted to clarify which signaling pathways were involved in regulation. These two enrichment analyses were implemented using clusterProfiler (14), and a false discovery rate (FDR) *p* < 0.25 was used as the standard for the statistical difference between the two enrichment analyses. Alternatively, Metascape (15) screening conditions were used, with statistical differences identified by *p* < 0.05, a minimum count of 3, and an enrichment factor > 1.

### GSEA

GSEA is an enrichment analysis method used to determine whether a set of a priori defined genes shows statistically significant and consistent differences between two biological states. According to the expression of lncRNA MSC-AS1, samples were divided into the high-expression group (>0.5) and the low-expression group (< 0.5), and the influence of the expression of lncRNA MSC-AS1 on other gene sets was analyzed accordingly. GSEA was carried out by using R packets clusterProfiler (3.8.0) (14). The number of random combinations was set at 1,000 times, and the significantly enriched gene sets were screened according to the criteria of an FDR *q*-value < 0.25 and an adjusted *p* < 0.05.

### Analysis of Immune Infiltration

Quantitative analysis of immune infiltration was carried out by single-sample GSEA (ssGSEA) with the GSVA package (16). The 24 types of immune cells in the tumor included neutrophils, mast cells, eosinophils, macrophages, natural kill (NK) cells, CD56dim NK cells, CD56bright NK cells, central memory CD4+ T cells (Tcms), dendritic cells (DCs), activated DCs (aDCs), plasmacytoid DCs (pDCs), CD8+ T cells, T helper cells, T cells, Th1 cells, Th2 cells, Th17 cells, T follicular helper cells (Tfhs), immature DCs (iDCs), Tregs, effector memory T cells (Tems), γδ T cells (Tgds), cytotoxic cells, and B cells (16). The Spearman correlation was used to analyze the correlation between single genes and relative infiltration richness/enrichment (enrichment score) of these 24 types of cells. The Wilcoxon rank sum test analyzed the relationship between the level of lncRNA MSC-AS1 expression or different clinicopathological factors and the infiltration of immune cells (enrichment score). We also explored the correlation between lncRNA MSC-AS1 and cancer immune infiltrates using CIBERSORT which is a deconvolution algorithm based on gene expression (17) (http://cibersort.stanford.edu/).

### Protein-Protein Interaction Analysis

Search Tool for the Retrieval of Interacting Genes (STRING) is an online database that searches for known proteins and predicts protein interaction relationships, including direct physical interactions between proteins and indirect functional correlations. The STRING database collects, evaluates, and integrates all publicly available protein-protein interaction information and complements this information with computational predictions to build a protein-protein interaction network (18). The software analyzes all DEGs; the interaction score threshold was set at 0.7.

### Statistical Analysis

Normal/correction, Pearson χ2, Fisher exact, and univariate logistic regression tests were used to analyze the correlation between the level of clinicopathological factors and the level of lncRNA MSC-AS1 expression. For the collected clinicopathological data, univariate Cox analysis was adopted, and *p* < 0.1 was included in the multivariate Cox analysis. The median value of lncRNA MSC-AS1 expression was set as the threshold, according to which patients were divided into a high-risk group and a low-risk group, and the survival curve was plotted by the Kaplan-Meier method and tested with the log-rank test (*p* < 0.01). Clinicopathological data included gender, race, age, histological type, residual tumor, histological grade, anatomic neoplasm subdivision, reflux history, antireflux treatment, Barrett's esophagus, *TP53* status, *PIK3CA* status, T stage (depth of invasion), N stage (lymph node metastasis), M stage, and TNM stage (according to the 8th edition of the American Joint Committee on Cancer's TNM cancer staging system). For all tests, *p*-values were two sided and *p* < 0.05 was considered statistically significant. All statistical analyses were carried out using R (3.6.3). The receiver operating characteristic (ROC) curve was used to quantitatively evaluate the efficacy of lncRNA MSC-AS1 expression values in differentiating tumor from normal samples using the pROC package (19). An area under the curve (AUC) value between 0.5 and 0.7 was of low accuracy; between 0.7 and 0.9, of medium accuracy; and above 0.9, of high accuracy.

### Model Building and Evaluation

Using the independent prognostic factors obtained from multiple factors and according to the multivariate Cox regression model, the RMS package (version 5.1-3; http://cran.rproject.org/w-eb/packages/rms/index.html) was used to plot the nomogram. From the original data, 1,000 samples were randomly sampled to form the internal data set for verification, and the data set was used to line up the internal part of the line graph for verification. The C-index was used to evaluate the pretesting capability of the module, and a calibration plot was used to determine the accuracy of the pretesting character. The calibration reflected indicated the prediction efficiency of the model, indicating whether Cox prognostic models such as overall survival and disease-free survival were good at predicting survival of patients. The calibration plot was a comparison between the premeasured risk and the actual risk of the patient. The closer the premeasured risk was to the standard curve, the better the compliance of the model. The C-index was obtained by ROC analysis of the risk score of the multivariate Cox model of the survival state, and it was used to quantify the prognostic evaluation efficacy of the tumor prognosis model.

## Results

### Clinical Characteristics

The characteristics of patients with gastric adenocarcinoma in TCGA—namely, gender, race, age, and so on—were collected. According to the mean expression of lncRNA MSC-AS1, 187 patients were assigned to the high-expression group, and 188 patients were assigned to the low-expression group. The χ^2^ test or Fisher's exact test determined that lncRNA MSC-AS1 expression was significantly associated with T stage (*p* < 0.001), pathological stage (*p* = 0.002), race (*p* = 0.015), histological type (*p* < 0.001), *TP53* status (*p* = 0.005), and *PIK3CA* status (*p* = 0.046). No correlation existed between lncRNA MSC-AS1 expression and the other clinicopathological features, as shown in Table 1.

**Table 1:** Association between long non-coding RNA (lncRNA) musculin antisense RNA1 (MSC-AS1) expression and clinicopathological features in the validation cohort.

**Characters**	**level**	**Low expression of MSC-AS1**	**High expression of MSC-AS1**	***P***	**Test**
*N*		188	187		
T stage (%)	T1	17 (9.0%)	2 (1.1%)	<0.001	exact
	T2	48 (25.5%)	32 (17.9%)		
	T3	83 (44.1%)	85 (47.5%)		
	T4	40 (21.3%)	60 (33.5%)		
N stage (%)	N0	60 (33.0%)	51 (29.1%)	0.370	exact
	N1	52 (28.6%)	45 (25.7%)		
	N2	39 (21.4%)	36 (20.6%)		
	N3	31 (17.0%)	43 (24.6%)		
M stage (%)	M0	166 (92.7%)	164 (93.2%)	1.000	exact
	M1	13 (7.3%)	12 (6.8%)		
Pathologic stage (%)	Stage I	39 (21.7%)	14 (8.1%)	0.002	exact
	Stage II	51 (28.3%)	60 (34.9%)		
	Stage III	68 (37.8%)	82 (47.7%)		
	Stage IV	22 (12.2%)	16 (9.3%)		
Primary therapy outcome (%)	CR	116 (71.6%)	115 (74.2%)	0.669	exact
	PD	36 (22.2%)	29 (18.7%)		
	PR	1 (0.6%)	3 (1.9%)		
	SD	9 (5.6%)	8 (5.2%)		
Gender (%)	Female	68 (36.2%)	66 (35.3%)	0.914	exact
	Male	120 (63.8%)	121 (64.7%)		
Race (%)	Asian	40 (26.3%)	34 (19.9%)	0.015	exact
	Black or African American	9 (5.9%)	2 (1.2%)		
	White	103 (67.8%)	135 (78.9%)		
Age (%)	≤ 65	76 (41.1%)	88 (47.3%)	0.251	exact
	>65	109 (58.9%)	98 (52.7%)		
Histological type (%)	Diffuse type	22 (11.7%)	41 (22.0%)	NA	exact
	Mucinous type	4 (2.1%)	15 (8.1%)		
	Not otherwise specified	104 (55.3%)	103 (55.4%)		
	Papillary type	3 (1.6%)	2 (1.1%)		
	Signet ring type	4 (2.1%)	7 (3.8%)		
	Tubular type	51 (27.1%)	18 (9.7%)		
Residual tumor (%)	R0	157 (91.3%)	141 (89.8%)	0.603	exact
	R1	6 (3.5%)	9 (5.7%)		
	R2	9 (5.2%)	7 (4.5%)		
Histologic grade (%)	G1	6 (3.3%)	4 (2.2%)	<0.001	exact
	G2	92 (50.0%)	45 (24.7%)		
	G3	86 (46.7%)	133 (73.1%)		
Anatomic neoplasm subdivision (%)	Antrum/distal	66 (36.3%)	72 (40.2%)	0.064	exact
	Cardia/proximal	24 (13.2%)	24 (13.4%)		
	Fundus/body	61 (33.5%)	69 (38.5%)		
	Gastroesophageal junction	27 (14.8%)	14 (7.8%)		
	Other	4 (2.2%)	0(0.0%)		
Reflux history (%)	No	96 (80.7%)	79 (83.2%)	0.723	exact
	Yes	23 (19.3%)	16 (16.8%)		
Antireflux treatment (%)	No	72 (78.3%)	70 (80.5%)	0.854	exact
	Yes	20 (21.7%)	17 (19.5%)		
Barretts esophagus (%)	No	113 (91.1%)	80 (95.2%)	0.291	exact
	Yes	11 (8.9%)	4 (4.8%)		
TP53 status (%)	Mut	100 (53.5%)	72 (38.9%)	0.005	exact
	WT	87 (46.5%)	113 (61.1%)		
PIK3CA status (%)	Mut	37 (19.8%)	22 (11.9%)	0.046	exact
	WT	150 (80.2%)	163 (88.1%)		
Age (median [IQR])		68.00 [59.00, 74.00]	66.50 [57.00, 72.75]	0.125	Non-norm

### High Expression of LncRNA MSC-AS1 in Gastric Tissues

**Figure 1:**
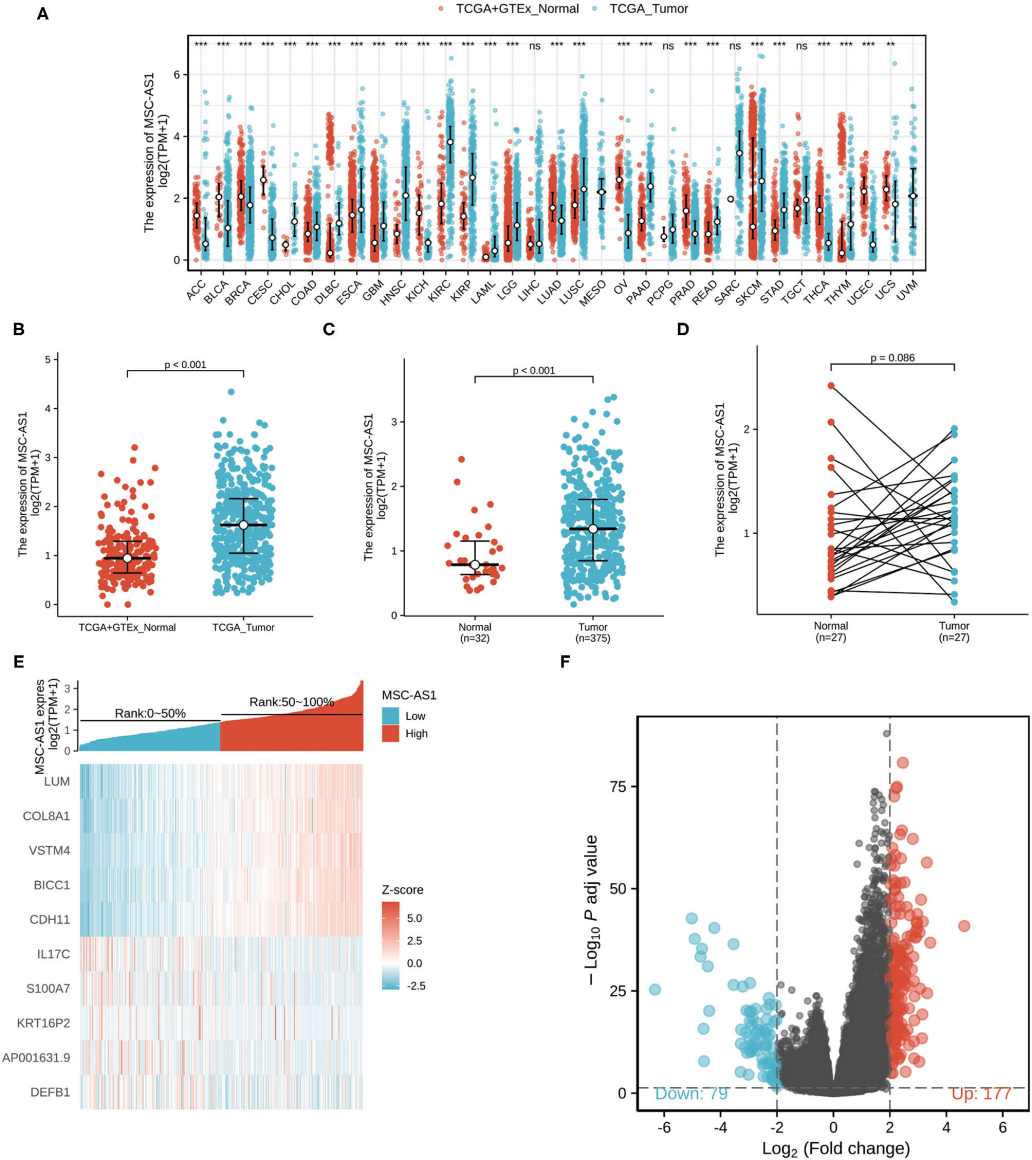
The expression of long non-coding RNA MSC-AS1 in **(A)** in Genotype-Tissue Expression (GTEx) and normal The Cancer Genome Atlas (TCGA) samples with corresponding TCGA tumor samples; **(B)** in GTEx and normal TCGA samples with TCGA gastric cancer gastric cancer (STAD) samples; **(C)** in 375 gastric cancer tissues and 32 normal tissues in TCGA database; and **(D)** in 27 pairs of gastric cancer tissues and non-cancerous adjacent tissues in TCGA database; **(E)** Volcano plot of differentially expressed long non-coding RNAs (lncRNAs). Normalized expression levels are shown in descending order from green to red. There 177 differential molecules had log2FC > 2 and adjusted *p* < 0.05, and 79 differential molecules had log2FC < −2 and adjusted *p* < 0.05. **(F)** Heat map of the 10 differentially expressed long non-coding RNAs (lncRNAs). The X-axis represents the expression of lncRNA MSC-AS1, while the Y-axis denotes different the differentially expressed lncRNAs. Green and red tones represent downregulated and upregulated lncRNAs, respectively.

Downloaded RNA-seq data in TPM format from TCGA and Genotype-Tissue Expression (GTEx) was processed uniformly using the Toil process from XENA (https://xenabrowser.net/datapages/) by the University of California, Santa Cruz (20). As seen in Figure 1A, the Wilcoxon rank sum test was used to compare the expression of MSC-AS1 in GTEx and normal TCGA samples with corresponding TCGA tumor samples. MSC-AS1 was significantly expressed in adrenal cortical carcinoma (ACC), bladder urothelial carcinoma (BLCA), breast-infiltrating carcinoma (BRCA), cervical squamous carcinoma and adenocarcinoma (CESC), bile duct carcinoma (CHOL), colon cancer (COAD), diffuse large B-cell lymphoma (DLBC), esophageal cancer (ESCA), lung adenocarcinoma (LUAS), lung squamous carcinoma (LUSC), ovarian serous cystadenocarcinoma (OV), pancreatic cancer (PAAD), prostate cancer (PRAD), rectal adenocarcinoma (READ), skin melanoma (SKCM), gastric cancer (STAD), and other cancers, and the results were statistically significant. The Wilcoxon rank sum test was used to compare the expression of MSC-AS1 in GTEx and normal TCGA samples with TCGA gastric cancer gastric cancer (STAD) samples (Figure 1B). MSC-AS1 was highly expressed in STAD samples of gastric cancer, and results were statistically significant (*p* < 0.001). lncRNA MSC-AS1 expression was then analyzed in 375 gastric cancer tissues and in 32 normal tissues in the TCGA database using the Wilcoxon rank sum test. LncRNA MSC-AS1 showed significantly higher expression in cancer tissues than in normal tissues (*p* < 0.001) (Figure 1C). The expression of lncRNA MSC-AS1 in 27 pairs of gastric cancer tissues and non-cancerous adjacent tissues was also examined by applying the Wilcoxon signed-rank test; no significant difference was found in expression of lncRNA MSC-AS1 in STAD samples of gastric cancer (*p* = 0.086) (Figure 1D).

### Identification of DEGs

The qualifying HTSeq-counts format data were divided into high- and low-expression groups based on the cutoff criteria according to the expression of lncRNA MSC-AS1 in the tumor sample. Then, 256 DEGs were obtained using the DESeq2 package. |log2FC| > 2 and adjusted *p* < 0.01 were used as the screening threshold for the DEGs. Among them, 177 were upregulated, and 79 were downregulated (Figure 1E, Supplementary Table 1). Then, DEGs in HTSeq-Counts were further analyzed by DESeq2 package. Relative expression values of the top 10 DEGs between the two cohorts were showed in Figure 1F.

### Functional Enrichment Analysis of DEGs

**Figure 2:**
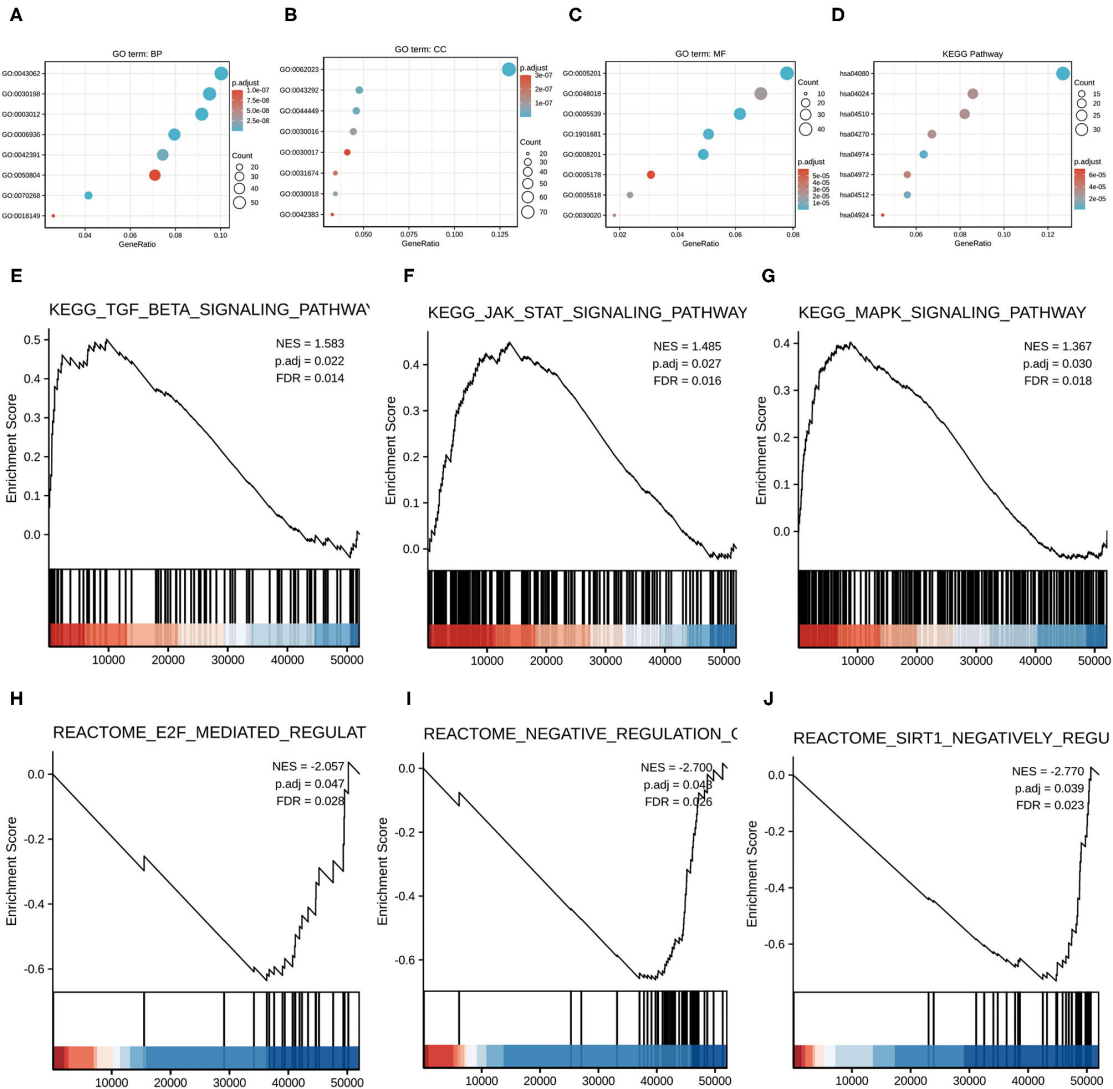
Functional enrichment analysis of 256 differentially expressed genes (DEGs) between high and low expression of lncRNA MSC-AS1 in patients with gastric cancer in TCGA. **(A)** Enriched Gene Ontology (GO) terms in the biological process category. **(B)** Enriched GO terms in the cellular component category. **(C)** Enriched GO terms in the molecular function category. **(D)** Enriched GO terms in the Kyoto Encyclopedia of Genes and Genomes (KEGG) category. The X-axis represents the proportion of DEGs, and the Y-axis represents different categories. The different colors indicate different properties, and the different sizes represent the numbers of DEGs. **(E–J)** Enrichment plots from Gene Set Enrichment Analysis (GSEA). The TGF-β signaling pathway, the JAK-STAT signaling pathway, and the MAPK signaling pathway were differentially enriched in the long non-coding RNA (lncRNA) MSC-AS1 high-expression phenotype. In the lncRNA MSC-AS1 low-expression phenotype, enriched pathways included E2F mediated regulation of DNA replication, negative regulation of NOTCH4 signaling, and SIRT1 negatively regulates RNA expression.

To better analyze the function implications of lncRNA MSC-AS1 in gastric cancer from the 256 DEGs between low and high lncRNA MSC-AS1 expression, GO and KEGG functional enrichment analyses were applied using the clusterProfiler package (Supplementary Tables 2, 3). GO function analysis of differentially expressed genes was divided into three parts: biological process, cellular component, and molecular function. In the biological process section, categorization indicated 528 GO terms (Figures 2A–C). KEGG was used for enrichment analysis of DEGs, and the results showed that DEGs were mainly enriched in neuroactive ligand-receptor interactions (hsa04080), protein digestion and absorption (hsa04974), extracellular matrix (ECM) receptor interaction (hsa04512), vascular smooth muscle contraction (hsa04270), the Cyclic Adenosine 3′,5′-monophosphate (cAMP) signaling pathway (hsa04024), focal adhesion (hsa04510), pancreatic secretion (hsa04972), and renin secretion (hsa04924) (Figure 2D).

### LncRNA MSC-AS1 Related Signaling Pathways

Analyzing lncRNA MSC-AS1 related signaling pathways was based on the results of co-expression analysis of lncRNA MSC-AS1 using the STAD expression matrix of gastric cancer in TCGA. GSEA was performed on the low-expression and high-expression lncRNA MSC-AS1 groups using the clusterProfiler package, in which C2.all.v7.0.symbols.gmt [Curated] was selected from MSigDB Collections as the reference gene collection; an FDR *q*-value < 0.25 and an adjusted *p* < 0.05 were considered significantly enriched. A total of 1,074 data sets met the requirements of FDR < 0.25 and adjusted *p* < 0.05. This analysis revealed that, in the lncRNA MSC-AS1 high-expression phenotype, 770 pathways were significantly differentially enriched, including the transforming growth factor β (TGF-β) signaling pathway, the JAK-STAT signaling pathway, and the MAPK signaling pathway. In addition, 304 pathways in the lncRNA MSC-AS1 low-expression phenotype were recognized, including the transcription factor E2F-mediated regulation of DNA replication, negative regulation of NOTCH4 signaling, and SIRT1 negative regulation of RNA expression (Figures 2E–J, Supplementary Table 4).

**Figure 3:**
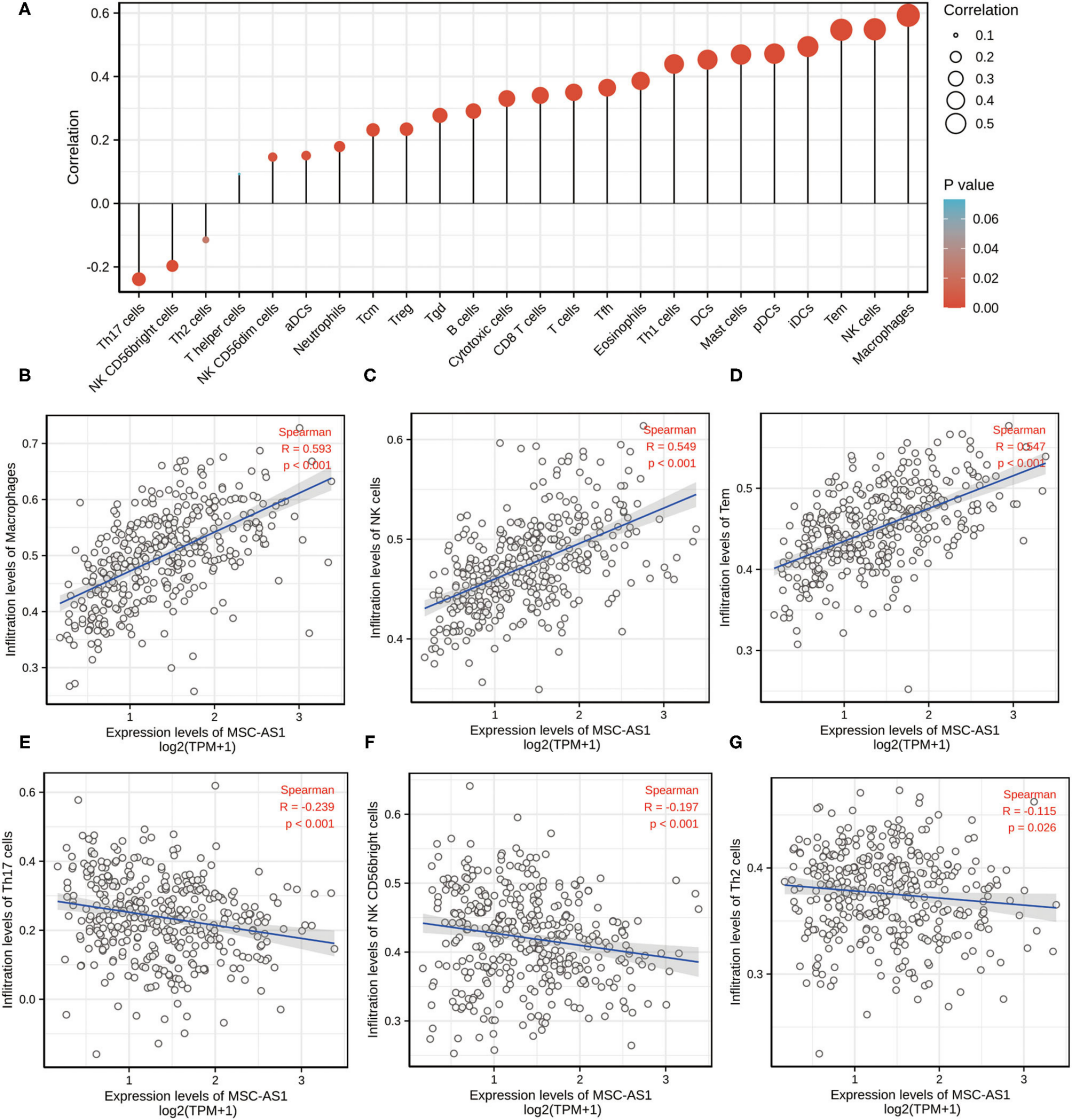
The expression level of long non-coding RNA (lncRNA) MSC-AS1 was associated with immune infiltration in the tumor microenvironment. **(A)** Correlation between the relative abundances of 24 immune cells and lncRNA MSC-AS1 expression level. The size of dots shows the absolute value of Spearman. Correlation between the relative enrichment score of Macrophages **(B)**, NK cells **(C)**, Tem (T effector memory) **(D)**, Th17 cells **(E)**, NK CD56bright cells **(F)**, Th2 cells **(G)** and the expression level (TPM) of lncRNA MSC-AS1.

Marker genes of 24 immune cells reflecting immune infiltration were extracted from the literature (10). Using the Spearman correlation, the relationship between the expression level (TPM) of lncRNA MSC-AS1 and the infiltration of the 24 immune cells in STAD of gastric cancer was analyzed with ssGSEA. LncRNA MSC-AS1 expression was significantly positively correlated with macrophages, natural killer (NK) cells, Tems, iDCs, and more. Helper T17 (Th17) cells, NK CD56bright cells, and Th2 cells were negatively correlated with lncRNA MSC-AS1 expression (*p* < 0.05). Macrophages were significantly positively correlated with lncRNA MSC-AS1 expression with a Spearman *r* ≤ 0.593 and *p* < 0.001. NK cells were significantly positively correlated with lncRNA MSC-AS1 expression with a Spearman *r* ≤ 0.549 and *p* < 0.001. T effector memory (Tem) were significantly positively correlated with lncRNA MSC-AS1 expression with Spearman r up to 0.547 with a *p*-value < 0.001. However, Th17 cells (R = −0.239, *P* < 0.001), NK CD56bright cells (R = −0.197, *P* < 0.001) and Th2 cells (R = −0.115, *P* = 0.026) showed a negative association with MSC-AS1 (Figures 3A–G). At the same time, we also applied CIBERSORT to analyze the correlation between lncRNA MSC-AS1 and cancer immune infiltrates (Supplementary Figure 1).

### Protein-Protein Interaction Enrichment Analysis

**Figure 4:**
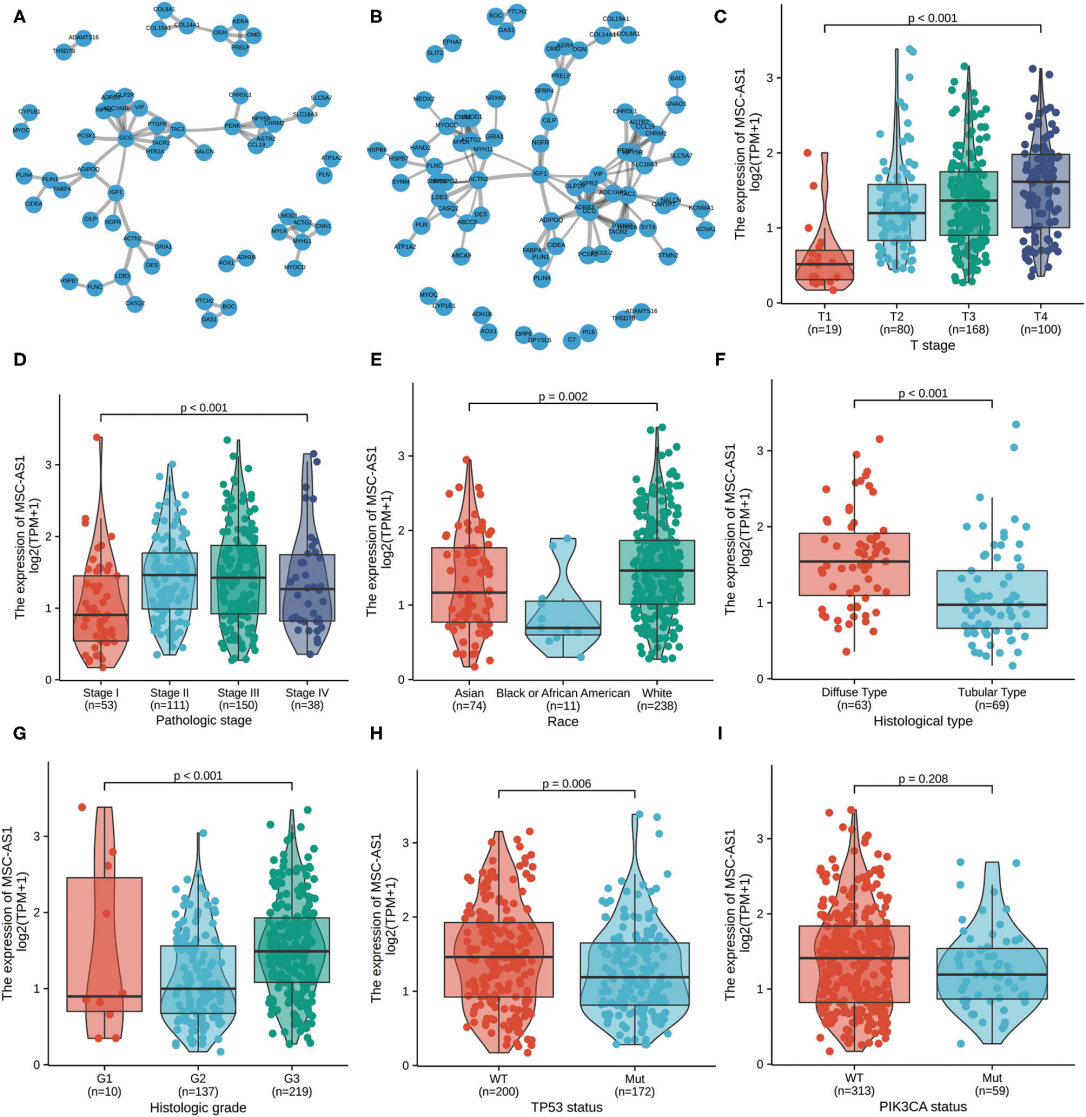
The protein-protein interaction (PPI) network of MSC-AS1 was constructed. A network of MSC-AS1 and its co-expression genes was set up visually. The interaction threshold was set to 0.7 **(A)** and 0.5 **(B)**. Association of long non-coding RNA (lncRNA) MSC-AS1 expression and clinicopathological characteristics: **(C)** T stage; **(D)** pathological stage; **(E)** race; **(F)** histological type; **(G)** histological grade; **(H)** *TP53* status; and **(I)** *PIK3CA* status.

To assess downregulated and upregulated DEGs, protein-protein interaction enrichment analysis was applied with the following three databases: BioGrid, InWeb, and OmniPath. The Molecular Complex Detection (MCODE) algorithm was carried out to discriminate densely connected network components, and the premise was that the network included between three and 500 proteins. The MCODE networks identified for the DEGs were compiled. The four most significant MCODE components, which had the four best-scoring terms by *p*-value, were retained; these represented the functional description of the corresponding components. After pathway and process enrichment analyses were independently carried out with every MCODE component, the results revealed that extracellular matrix organization, degradation of the extracellular matrix, integrin cell surface interactions, extracellular matrix proteoglycans, and formation of the cornified envelope and pathways in cancer. The interaction threshold was set to 0.7 (Figure 4A, Supplementary Table 5) and 0.5 (Figure 4B, Supplementary Table 6).

Supplementary Table 5, MSC-AS1 and its co-expression genes, the interaction threshold was set to 0.7.

Supplementary Table 6, MSC-AS1 and its co-expression genes, the interaction threshold was set to 0.5.

### Correlations Between LncRNA MSC-AS1 Expression and Clinical Characteristics in Patients With Gastric Cancer

Overall, 375 gastric cancer samples with lncRNA MSC-AS1 expression data were collected and analyzed from TCGA. Increased expression of lncRNA MSC-AS1 correlated significantly with T stage (*p* < 0.001), pathological stage (*p* = 0.002), race (*p* = 0.015), histological type (*p* < 0.001), histological grade (*p* < 0.001), *TP53* status (*p* = 0.005), and *PIK3CA* status (*p* = 0.046) using the Kruskal-Wallis test and the Wilcoxon signed-rank test, as shown in Figures 4C–I.

Univariate logistic regression revealed that the increased lncRNA MSC-AS1 expression was related to poor prognostic clinicopathological characteristics, including a greater primary tumor extent [odds ratio (OR) = 2.25; 95% CI, 1.40–3.67) for T3 and T4 stages vs. T1 and T2 stages (*p* < 0.001), more serious histological type (OR = 5.28; 95% CI, 2.54–11.37) for diffuse type vs. tubular type (*p* < 0.001), more advanced histological grade (OR = 3.09; 95% CI, 2.01–4.82) for grade 3 vs. grades 1 and 2 (*p* < 0.001), *TP53* status (OR = 0.55; 95% CI, 0.37–0.84) for mutated vs. wild type (*p* = 0.005), and *PIK3CA* status (OR = 0.55; 95% CI, 0.30–0.96) for mutated vs. wild type (*p* = 0.039) (Table 2). In addition, χ^2^ analysis confirmed these results between clinicopathological features and lncRNA MSC-AS1 expression. The results indicated that gastric cancer with increased lncRNA MSC-AS1 expression is prone to poor clinicopathological factors.

**Table 2:** Association of lncRNA MSC-AS1 expression with clinical pathological characteristics by logistic regression.

**Characteristics**	**Odds ratio in MSC-AS1 expression**	**Odds ratio (OR)**	***P*** **-value**
T stage (T3 and T4 vs. T1 and T2)	367	2.25 (1.40–3.67)	<0.001
N stage (N1 and N2 and N3 vs. N0)	357	1.20 (0.76–1.88)	0.435
M stage (M1 vs. M0)	355	0.93 (0.41–2.12)	0.870
Pathologic stage (Stage III and Stage IV vs. Stage I and Stage II)	352	1.32 (0.87–2.02)	0.190
Histological type (diffuse type vs. tubular type)	132	5.28 (2.54–11.37)	<0.001
Histologic grade (G3 vs. G1 and G2)	366	3.09 (2.01–4.82)	<0.001
Primary therapy outcome (CR vs. PD and SD and PR)	317	1.14 (0.69–1.88)	0.604
Residual tumor (R1 and R2 vs. R0)	329	1.19 (0.56–2.51)	0.649
TP53 status (Mut vs. WT)	372	0.55 (0.37–0.84)	0.005
PIK3CA status (Mut vs. WT)	372	0.55 (0.30–0.96)	0.039

### ROC Differentiates Normal Tissue From Tumor Tissue

**Figure 5:**
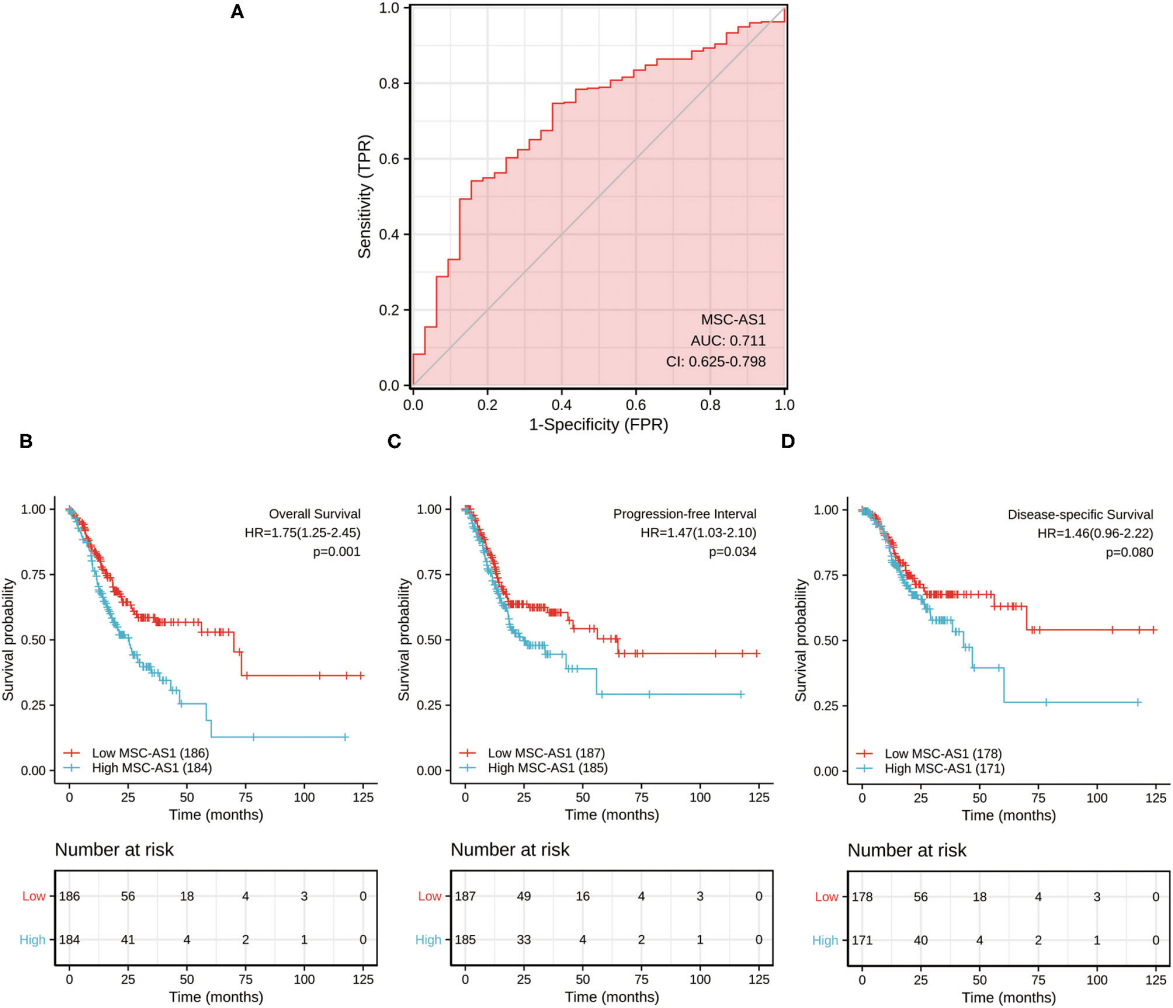
**(A)** Receiver operating characteristic (ROC) analysis of long non-coding RNA (lncRNA) MSC-AS1 expression showing promising discrimination power between non-tumor and tumor tissues. The area under the curve (AUC) is plotted as sensitivity (%) vs. 100% specificity. Impact of long non-coding RNA (lncRNA) MSC-AS1 expression on **(B)** overall survival, **(C)** progression-free interval, and **(D)** disease-specific survival in patients with gastric cancer in The Cancer Genome Atlas (TCGA) cohort.

The data from para-carcinoma tissue of patients and carcinoma tissue of patients were applied to draw the ROC curve and evaluate the diagnostic value of lncRNA MSC-AS1. Its AUC was 0.711, predicting a very efficient discrimination value for gastric cancer (Figure 5A).

### Role of LncRNA MSC-AS1 in Gastric Cancer Survival

Kaplan-Meier survival analysis showed that a high expression level of lncRNA MSC-AS1 was significantly correlated with a poorer 10-year overall survival (OS) of patients [hazard ratio (HR) = 1.75; 95% confidence interval (CI), 1.25–2.45; *p* = 0.001) (Figure 5B). Patients with high lncRNA MSC-AS1 expression had significantly poorer 10-year progression-free interval (PFI) than did those with low lncRNA MSC-AS1 expression (HR = 1.47; 95% CI, 1.03–2.10; *p* = 0.034) (Figure 5C). However, no correlation existed between the expression level of MSC-AS1 and the outcome of 10-year disease-specific survival (DSS) (HR = 1.46; 95% CI, 0.96-2.22; *p* = 0.080) (Figure 5D).

Univariate analysis assessed the prognostic factors for overall survival with the Cox regression model. Patients with high lncRNA MSC-AS1 expression were associated with significantly poorer overall survival (OS) (HR = 1.75; 95% CI, 1.25–2.45; *p* = 0.001). Factors that were clearly related to overall survival also included T stage for T3 and T4 vs. T1 and T2 (HR = 1.71; 95% CI, 1.13–2.61; *p* = 0.011), N stage for N1, N2, and N3 vs. N0 (HR = 1.92; 95% CI, 1.26–2.93; *p* = 0.002), M stage for M1 vs. M0 (HR = 2.25; 95% CI, 1.29–3.92; *p* = 0.004), pathological stages III and IV vs. Stages I and II (HR = 1.94; 95% CI, 1.35–2.79; *p* < 0.001), primary therapy outcome for complete response vs. progressive disease, stable disease, and partial response (HR = 0.24; 95% CI, 0.16–0.34; *p* < 0.001), residual tumor R1 and R2 vs. R0 (HR = 3.45; 95% CI, 2.16–5.49; *p* < 0.001), and age for >65 vs. ≤ 65 years (HR = 1.62; 95% CI, 1.15–2.77; *p* = 0.005) (Table 3). Multivariate analysis using Cox regression model was then performed. LncRNA MSC-AS1 expression level (*p* = 0.028), primary therapy outcome (*p* < 0.001), and age (*p* = 0.014) were independently correlated with overall survival in the multivariate analysis (Table 3). Univariate analysis also assessed the prognostic factors for disease-specific survival and progression-free interval with the Cox regression model. However, the increased lncRNA MSC-AS1 level was not related to poorer disease-specific survival or the progression-free interval (Tables 4, 5). These results indicate that lncRNA MSC-AS1 may have prognostic value and can be used as a biomarker for predicting the overall survival, disease-specific survival, and disease-free survival of patients with gastric cancer.

**Table 3:** Associations between overall survival and clinicopathological characteristics in patients in TCGA using Cox regression.

**Characteristics**	**Total (*N*)**	**HR (95% CI)**	***P*** **-value**	**HR (95% CI)**	***P*** **-value**
		**Univariate analysis**	**univariate analysis**	**multivariate analysis**	**multivariate analysis**
T stage (T3 and T4 vs. T1 and T2)	362	1.719 (1.131–2.612)	0.011	1.101 (0.590–2.054)	0.763
N stage (N1 and N2 and N3 vs. N0)	352	1.925 (1.264–2.931)	0.002	1.421 (0.672–3.006)	0.357
M stage (M1 vs. M0)	352	2.254 (1.295–3.924)	0.004	0.991 (0.382–2.572)	0.985
Pathologic stage (Stage III and Stage IV vs. Stage I and Stage II)	347	1.947 (1.358–2.793)	<0.001	1.285 (0.676–2.440)	0.444
Histologic grade (G3 vs. G1 and G2)	361	1.353 (0.957–1.914)	0.087	1.349 (0.840–2.167)	0.215
Histological type (diffuse type vs. tubular type)	132	1.077 (0.620–1.872)	0.793		
Primary therapy outcome (CR vs. PD and SD and PR)	313	0.237 (0.163-0.344)	<0.001	0.223 (0.142–0.350)	<0.001
Residual tumor (R1 and R2 vs. R0)	325	3.445 (2.160–5.494)	<0.001	1.307 (0.662–2.581)	0.441
Age (>65 vs. ≤ 65)	367	1.620 (1.154–2.276)	0.005	1.736 (1.118–2.698)	0.014
Race (Asian and Black or African American vs. White)	320	0.801 (0.515–1.247)	0.326		
Gender (male vs. female)	370	1.267 (0.891–1.804)	0.188		
Anatomic neoplasm subdivision (fundus/body vs. antrum/distal)	267	0.965 (0.651–1.430)	0.858		
Reflux history (yes vs. no)	213	0.582 (0.291–1.162)	0.125		
Antireflux treatment (yes vs. no)	179	0.756 (0.422–1.353)	0.346		
Barretts esophagus (yes vs. no)	207	0.892 (0.326-2.441)	0.824		
TP53 status (Mut vs. WT)	367	0.865 (0.621–1.205)	0.392		
PIK3CA status (Mut vs. WT)	367	0.623 (0.370–1.048)	0.075	0.675 (0.370–1.234)	0.202
MSC-AS1 (high vs. low)	370	1.753 (1.254–2.451)	0.001	1.681 (1.057–2.673)	0.028

**Table 4:** Associations between disease-specific survival and clinicopathological characteristics in patients in TCGA using Cox regression.

**Characteristics**	**Total (*N*)**	**HR (95% CI)**	***P*** **-value**	**HR (95% CI)**	***P*** **-value**
		**Univariate analysis**	**univariate analysis**	**multivariate analysis**	**multivariate analysis**
T stage (T3 and T4 vs. T1 and T2)	345	2.089 (1.192–3.660)	0.010	1.052 (0.529–2.092)	0.884
N stage (N1 and N2 and N3 vs. N0)	334	1.807 (1.075–3.036)	0.025	0.802 (0.325–1.978)	0.632
M stage (M1 vs. M0)	333	2.438 (1.221–4.870)	0.012	0.888 (0.350–2.251)	0.802
Pathologic stage (Stage III and Stage IV vs. Stage I and Stage II)	331	2.146 (1.352–3.404)	0.001	1.703 (0.776–3.737)	0.184
Histologic grade (G3 vs. G1 and G2)	340	1.338 (0.862–2.078)	0.194		
Histological type (diffuse type vs. tubular type)	129	1.115 (0.597–2.082)	0.734		
Primary therapy outcome (CR vs. PD and SD and PR)	310	0.115 (0.072–0.184)	<0.001	0.108 (0.061–0.192)	<0.001
Residual tumor (R1 and R2 vs. R0)	314	5.142 (3.014–8.771)	<0.001	1.989 (1.036–3.816)	0.039
Age (>65 vs. ≤ 65)	346	1.211(0.797–1.840)	0.371		
Race (Asian and Black or African American vs. White)	305	1.097 (0.656–1.836)	0.724		
Gender (male vs. female)	349	1.573(0.985–2.514)	0.058	1.543 (0.874–2.722)	0.135
Anatomic neoplasm subdivision (fundus/body vs. antrum/distal)	253	0.850 (0.512–1.412)	0.531		
Reflux history (yes vs. no)	208	0.598 (0.272–1.313)	0.200		
Antireflux treatment (yes vs. no)	167	0.758 (0.380–1.511)	0.431		
Barretts esophagus (yes vs. no)	201	0.974 (0.304–3.118)	0.964		
TP53 status (Mut vs. WT)	346	1.007 (0.662–1.532)	0.974		
PIK3CA status (Mut vs. WT)	346	0.815 (0.452–1.470)	0.497		
MSC-AS1 (high vs. low)	349	1.455 (0.956–2.216)	0.080	1.368 (0.816–2.295)	0.235

**Table 5:** Associations between progression-free interval and clinicopathological characteristics in patients in TCGA using Cox regression.

**Characteristics**	**Total (*N*)**	**HR (95% CI)**	***P*** **-value**	**HR (95% CI)**	***P*** **-value**
		**Univariate analysis**	**univariate analysis**	**multivariate analysis**	**multivariate analysis**
T stage (T3 and T4 vs. T1 and T2)	364	1.705 (1.095–2.654)	0.018	0.633 (0.315–1.274)	0.200
N stage (N1 and N2 and N3 vs. N0)	354	1.640 (1.075–2.501)	0.022	0.985 (0.398–2.437)	0.974
M stage (M1 vs. M0)	353	2.224 (1.194–4.144)	0.012	0.971 (0.324–2.910)	0.959
Pathologic stage (Stage III and Stage IV vs. Stage I and Stage II)	349	1.676 (1.154–2.435)	0.007	1.054 (0.431–2.582)	0.908
Histologic grade (G3 vs. G1 and G2)	363	1.540 (1.057–2.245)	0.025	1.460 (0.841–2.535)	0.179
Histological type (diffuse type vs. tubular type)	132	1.241 (0.719–2.144)	0.438		
Primary therapy outcome (CR vs. PD and SD and PR)	315	0.124 (0.085–0.183)	<0.001	0.090 (0.047–0.169)	<0.001
Residual tumor (R1 and R2 vs. R0)	326	3.469 (2.127–5.656)	<0.001	1.347 (0.658–2.755)	0.415
Age (>65 vs. ≤ 65)	369	0.858 (0.603–1.221)	0.395		
Race (Asian and Black or African American vs. White)	322	1.061 (0.688–1.637)	0.787		
Gender (male vs. female)	372	1.638 (1.099–2.440)	0.015	1.228 (0.625–2.412)	0.551
Anatomic neoplasm subdivision (fundus/body vs. antrum/distal)	267	0.728 (0.470–1.128)	0.156		
Reflux history (yes vs. no)	214	0.482 (0.232–1.000)	0.050	0.958 (0.324–2.832)	0.938
Antireflux treatment (yes vs. no)	179	0.584 (0.298–1.146)	0.118		
Barretts esophagus (yes vs. no)	208	0.953 (0.348–2.612)	0.926		
TP53 status (Mut vs. WT)	369	1.061 (0.744–1.514)	0.743		
PIK3CA status (Mut vs. WT)	369	0.895 (0.549–1.460)	0.657		
MSC–AS1 (high vs. low)	372	1.470 (1.029–2.100)	0.034	1.480 (0.832–2.631)	0.182

### Nomogram

**Figure 6:**
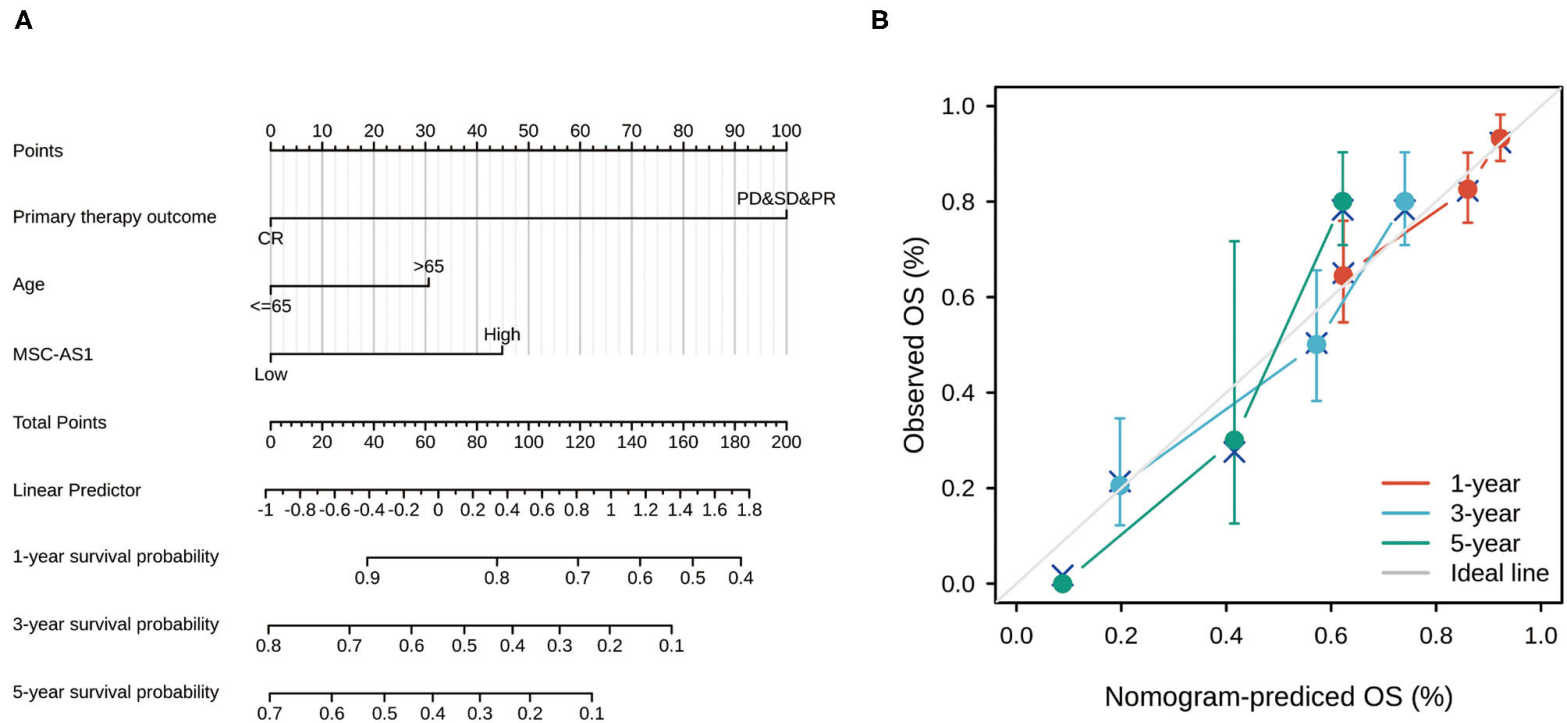
**(A)** Nomogram for predicting the probability of 1-, 3-, and 5-year overall survival (OS) for patients with gastric cancer. **(B)** Calibration plot of nomogram for predicting the probability of OS at 1, 3, and 5 years. The abscissa was the probability of the prognosis predicted by the model (0-1: the probability of the event occurring is 0–100%), and the ordinate was the actual observed prognosis. The colored line was the fit line and represented the predicted value (the horizontal axis) corresponding to the actual value (the vertical axis). The gray diagonal was the ideal case.

Univariate and multivariate Cox regression analyses identified three independent predictors—MSC-AS1 expression level, primary therapy outcome, and age—that were used to draw a nomogram and predict the prognosis of gastric cancer (Figure 6A). The corresponding line segment of each variable was marked with a scale, which represented the value range of the variable, and the length of the line segment reflected the contribution of this factor to the prognosis. The value on each prediction indicator scale corresponded to the score on the scoring scale. The score of all indicators was added to the total score, which corresponded to the predicted value of overall survival. The C-index of the model was 0.710 (95% CI, 0.685–0.734). The consistency between the premeasured value of the nomogram and the real observation value was shown, and the nomogram had high accuracy. The bootstrap method, self-sampling for 1,000 times, was used for internal verification of the nomogram prediction model, and then a calibration plot was drawn (Figure 6B). The results showed that the premeasured values of 1, 2, and 3 years of viability were close to the actual values and had good degrees of coincidence.

### Prognostic Performance of MSC-AS1 in Clinicopathological Subgroups

**Figure 7:**
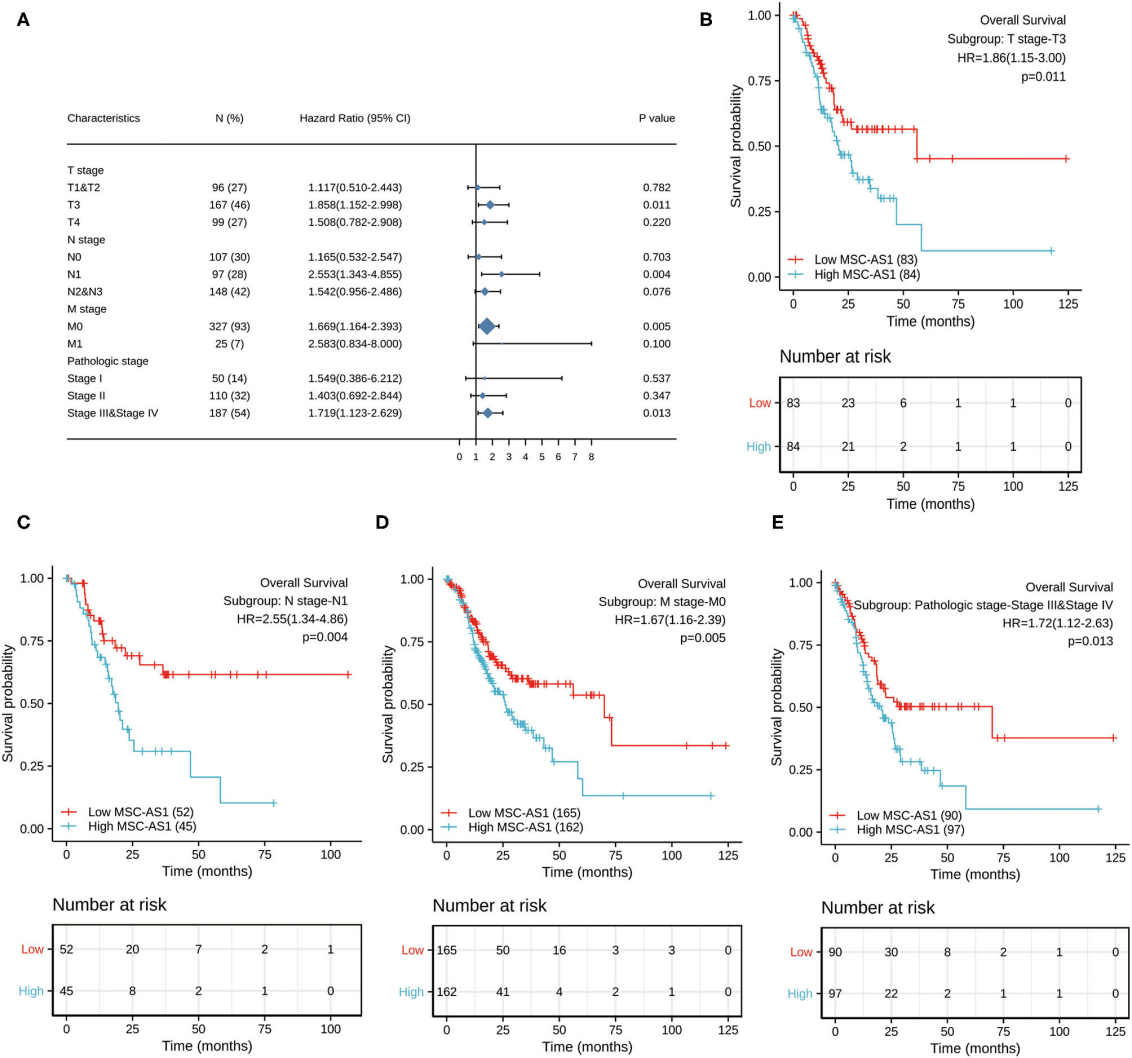
**(A)** The prognostic value of MSC-AS1 in TCGA STAD subsets of gastric cancer in The Cancer Genome Atlas. Statistically significant subgroups were **(B)** T3; **(C)** N1; **(D)** M0; and **(E)** pathological stages III and IV. TCGA, The Cancer Genome Atlas; GC, gastric cancer.

Next, we conducted subgroup survival analyses of OS, PFI and DSS, which showed that the prognosis of patients with MSC-AS1-high was poor in T3, N1, M0, and stage III-IV subgroups of OS. However, there was no significant difference in survival among each subgroup of DSS and PFI. The prognostic value for OS of MSC-AS1 in STAD subsets of TCGA gastric cancer was analyzed (Table 6, Figure 7A). In these subsets, the T3 subgroup of MSC-AS1 for the T stage was statistically significant (HR = 1.858; 95% CI, 1.152–2.998; *p* = 0.011) (Figure 7B), the N1 subgroup for the N stage was statistically significant (HR = 2.553; 95% CI, 1.343–4.855; *p* = 0.004) (Figure 7C), and the M0 subgroup for the M stage was statistically significant (HR = 1.669; 95% CI, 1.164–2.393; *p* = 0.005) (Figure 7D). Furthermore, the subgroups of pathological stages III and IV had statistical significance (HR = 1.719; 95% CI, 1.123–2.629; *p* = 0.013) (Figure 7E).

**Table 6:** Prognostic value for OS of MSC-AS1 for overall survival of STAD subgroups of gastric cancer in TCGA.

**Characteristics**	***N*** **(%)**	**HR (95% CI)**	***P*** **-value**
T stage			
T1 and T2	96 (27)	1.117 (0.510–2.443)	0.782
T3	167 (46)	1.858 (1.152–2.998)	0.011
T4	99 (27)	1.508 (0.782–2.908)	0.220
N stage			
N0	107 (30)	1.165 (0.532–2.547)	0.703
N1	97 (28)	2.553 (1.343–4.855)	0.004
N2 and N3	148 (42)	1.542 (0.956–2.486)	0.076
M stage			
M0	327 (93)	1.669 (1.164–2.393)	0.005
M1	25 (7)	2.583 (0.834–8.000)	0.100
Pathologic stage			
Stage I	50 (14)	1.549 (0.386–6.212)	0.537
Stage II	110 (32)	1.403 (0.692–2.844)	0.347
Stage III and Stage IV	187 (54)	1.719 (1.123–2.629)	0.013

## Discussion

Recently, the understanding of lncRNAs has evolved to identify a new viewpoint about their involvement in pathogenesis of disease. LncRNAs regulate gene expression through a variety of mechanisms, such as interactions with RNA or protein molecules. Currently, many lncRNAs have been confirmed as crucial biomarkers in stomach cancer.

Increasing numbers of studies have indicated that lncRNA MSC-AS1 plays an important role in some kinds of cancers by causing cancer cell proliferation, metastasis, and invasion and by accelerating the osteogenic differentiation in bone marrow stem cells via inhibition of miR-140-5p to induce BMP2 (8). One study revealed that MSC-AS1 exacerbated NPC progression by regulating the miR-524-5p/NR4A2 axis; therefore, lncRNA MSI- AS1 could promote the proliferation of NPC cells, inhibit cell apoptosis, and induce cell invasion and differentiation (12). Another study showed that MSC-AS1 promoted the occurrence of hepatocellular carcinoma via upregulation of the expression level of *PGK1* (11). Yet another study confirmed that MSC-AS1 promoted KIRC cell proliferation and migration via the miR3924/WNT5A/β-catenin axis (9). However, a correlation between lncRNA MSC-AS1 and stomach cancer has rarely been explored in the literature. This study aimed to clarify the expression level of lncRNA MSC-AS1 in stomach cancer tissues and identify its potential therapeutic and prognostic value.

In this study, we collected and organized stomach cancer data using high-throughput RNA sequencing from TCGA database, and we verified that lncRNA MSC-AS1 was significantly upregulated in stomach cancer tissues compared with in adjacent normal or normal tissues. Moreover, analyzing the relationship between the clinicopathological features of gastric cancer and the dichotomy of high and low MSC-AS1 levels by using the logistic regression method, we showed that MSC-AT1 was also significantly correlated with histological type, *TP53* status, and *PIK3CA* status. Upregulated lncRNA MSC-AS1 in stomach cancer tissues was positively correlated with higher T stage; advanced histological grade; and poorer prognosis, including poorer overall survival and progression-free survival. Elevated lncRNA MSC-AS1 was related to advanced clinicopathological features. These results suggest that lncRNA MSC-AS1 might be an independent biomarker of poor outcomes for stomach cancer.

We also investigated the function of lncRNA MSC-AS1 in stomach cancer tissues using GSEA, and the results showed that, in the high lncRNA MSC-AS1 expression phenotype, pathways such as the TGF-β signaling pathway, the JAK-STAT signaling pathway, the MAPK signaling pathway, E2F-mediated regulation of DNA replication, negative regulation of NOTCH4 signaling, and SIRT1 negative regulation of RNA expression were significantly differentially enriched. TGF-β mediates a wide range of biological activities, such as differentiation, epithelial cell growth, migration, extracellular matrix production, senescence, and angiogenesis (21, 22). We have previously shown that TGF-β was upregulated in peritoneal diffusion in hepatocellular carcinoma (23, 24). TGF-β also plays a crucial role in mesothelial cell senescence. In addition, epithelial mesenchymal transition (EMT) is driven by TGF-β and plays important roles in the metastasis of cancer. Recent studies showed that strong phosphorylated P38/MAPK in colorectal cancer was an independent prognostic factor, which predicted poorer survival. Angiogenesis is a necessary step in tumor metastasis, and vascular endothelial growth factor (VEGF) is a well-known angiogenesis factor. Inhibiting the VEGF pathway could lead to reduced colorectal cancer angiogenesis and decreased colorectal cancer proliferation and migration. All of these changes indicate that lncRNA MSC-AS1 might promote stomach cancer cell growth, metastasis, and poor survival via the MAPK and VEGF pathways. These pathways were confirmed as promoters of cancer cell proliferation, invasion, and metastasis, and these findings indicate the value of lncRNA MSC-AS1 as a new prognostic and therapeutic target in stomach cancer.

This study applied ssGSEA and Spearman correlation to reveal connections between lncRNA MSC-AS1 expression and immune infiltration levels in stomach cancer. We found that the relationships between lncRNA MSC-AS1 and macrophages, NK cells, Tems, and iDCs were the strongest. Moreover, we found a moderate to strong positive relationship between lncRNA MSC-AS1 and the infiltration level of some immune cells, particularly CD8 T cells, T cells, and cytotoxic cells. Conversely, levels of Th17 cells, NK CD56bright cells, and Th2 cells were negatively related to lncRNA MSC-AS1 expression. Thus, LncRNA MSC-AS1 likely plays a major role in immune cell infiltration and as a prognostic biomarker in stomach cancer.

To discover the molecular significance of MSC-AS1, coregulatory proteins were included in the PPI network analysis. LncRNA MSC-AS1 participates in the P38/MAPK pathway, the VEGF pathway, and so on, and we surmise that lncRNA MSC-AS1 may play an important role in the development and progression of stomach cancer by regulating these pathways.

To confirm the relationship between lncRNA MSC-AS1 and overall survival in stomach cancer, we adopted Kaplan-Meier survival analysis to the stratified clinicopathological characteristics. Kaplan-Meier survival analysis showed significant associations between the lncRNA MSC-AS1 expression level and overall survival with respect to T3, N1, M0, and stage IV disease, suggesting that the lncRNA MSC-AS1 expression level remains a strong predictor of prognosis in these subsets.

Although this study improved our knowledge about the association between lncRNA MSC-AS1 and stomach cancer, some limitations exist. First, to fully elucidate the special role of lncRNA MSC-AS1 in the development and progress of gastric cancer, all clinical factors, such as the details of patients' treatments, should be included. However, in a public database, such information is lacking or inconsistently processed. Second, this study provided only an analysis of bioinformation without experimental verification. Experiments such as quantitative polymerase chain reaction and immunohistochemical analysis are needed to study the function and mechanism of lncRNA MSC-AS1 in depth. Third, the understanding of gene function is not comprehensive with single omics, so extension to multiomics studies, especially the study of the protein level and its functional mechanism, should be performed. Fourth, the absence of an external dataset validation may result in bias. Last, a retrospective study has its own limitations; prospective studies must be carried out in the future.

In this study, we discovered that lncRNA MSC-AS1 is an independent predictor of poorer overall survival in stomach cancer. Moreover, our lncRNA MSC-AS1–related nomogram indicated that lncRNA MSC-AS1 contributed to overall survival more than age did.

## Data Availability Statement

The original contributions presented in the study are included in the article/Supplementary Material, further inquiries can be directed to the corresponding author/s.

## Ethics Statement

All data were collected and downloaded from the TCGA database. Since the TCGA database is made available to the public under specific guidelines, it can be confirmed that all written informed consent was given. Patients/participants provided written informed consent to participate in this study.

## Author Contributions

WY designed the ideas for this paper. WY, FG, YH, and YZ contributed to the writing of the manuscript. ZZ, BL, JC, LP, and SL contributed to data collation and data analysis. YZ, SL, ZS, and JH analyzed and interpreted the data. All authors contributed to this article and approved the submitted version.

## Funding

This study was supported by Henan Province Medical Science and Technology Tackling Program Joint Co-construction Project (No. LHGJ20200188).

## Conflict of Interest

The authors declare that the research was conducted in the absence of any commercial or financial relationships that could be construed as a potential conflict of interest.

## Publisher's Note

All claims expressed in this article are solely those of the authors and do not necessarily represent those of their affiliated organizations, or those of the publisher, the editors and the reviewers. Any product that may be evaluated in this article, or claim that may be made by its manufacturer, is not guaranteed or endorsed by the publisher.
