# Corrigendum: LncRNA MSC-AS1 Is a Diagnostic Biomarker and Predicts Poor Prognosis in Patients With Gastric Cancer by Integrated Bioinformatics Analysis

**DOI:** 10.3389/fmed.2022.862010

**Published:** 2022-02-17

**Authors:** Wei Yang, Fusheng Ge, Shuaibing Lu, Zhiming Shan, Liangqun Peng, Junhui Chai, Hongxing Liu, Baodong Li, Zhandong Zhang, Jinxi Huang, Yawei Hua, Yonglei Zhang

**Affiliations:** ^1^Department of General Surgery, The Affiliated Tumor Hospital of Zhengzhou University, Henan Cancer Hospital, Zhengzhou, China; ^2^Clinical Laboratory, The Children's Hospital of Henan Province, Zhengzhou Children's Hospital, The Affiliated Children Hospital of Zhengzhou University, Zhengzhou, China

**Keywords:** lncRNA MSC-AS1, prognosis, gastric cancer, bioinformatics, biomarker

Shuaibing Lu was not listed as a co-first author in the published article. The corrected author list appears below.

Wei Yang^1†^, Fusheng Ge^1†^, Shuaibing Lu^1†^, Zhiming Shan^2^, Liangqun Peng^1^, Junhui Chai^1^, Hongxing Liu^1^, Baodong Li^1^, Zhandong Zhang^1^, Jinxi Huang^1^, Yawei Hua^1*^ and Yonglei Zhang^1*^

In the published article, there was an error in affiliation 1. Instead of “Department of General Surgery, Henan Cancer Hospital, Affiliated Tumor Hospital of Zhengzhou University, Zhengzhou, China,” it should be “Department of General Surgery, The Affiliated Tumor Hospital of Zhengzhou University, Henan Cancer Hospital, Zhengzhou, China.”

The authors apologize for these errors and state that they do not change the scientific conclusions of the article in any way. The original article has been updated.

## Publisher's Note

All claims expressed in this article are solely those of the authors and do not necessarily represent those of their affiliated organizations, or those of the publisher, the editors and the reviewers. Any product that may be evaluated in this article, or claim that may be made by its manufacturer, is not guaranteed or endorsed by the publisher.

