# Supplementary figures and images for: LncRNA MSC-AS1 Is a Diagnostic Biomarker and Predicts Poor Prognosis in Patients With Gastric Cancer by Integrated Bioinformatics Analysis
Source: Front Med (Lausanne). 2021 Dec 2;8:795427. doi: 10.3389/fmed.2021.795427 (PMC8674534; doi:10.3389/fmed.2021.795427)

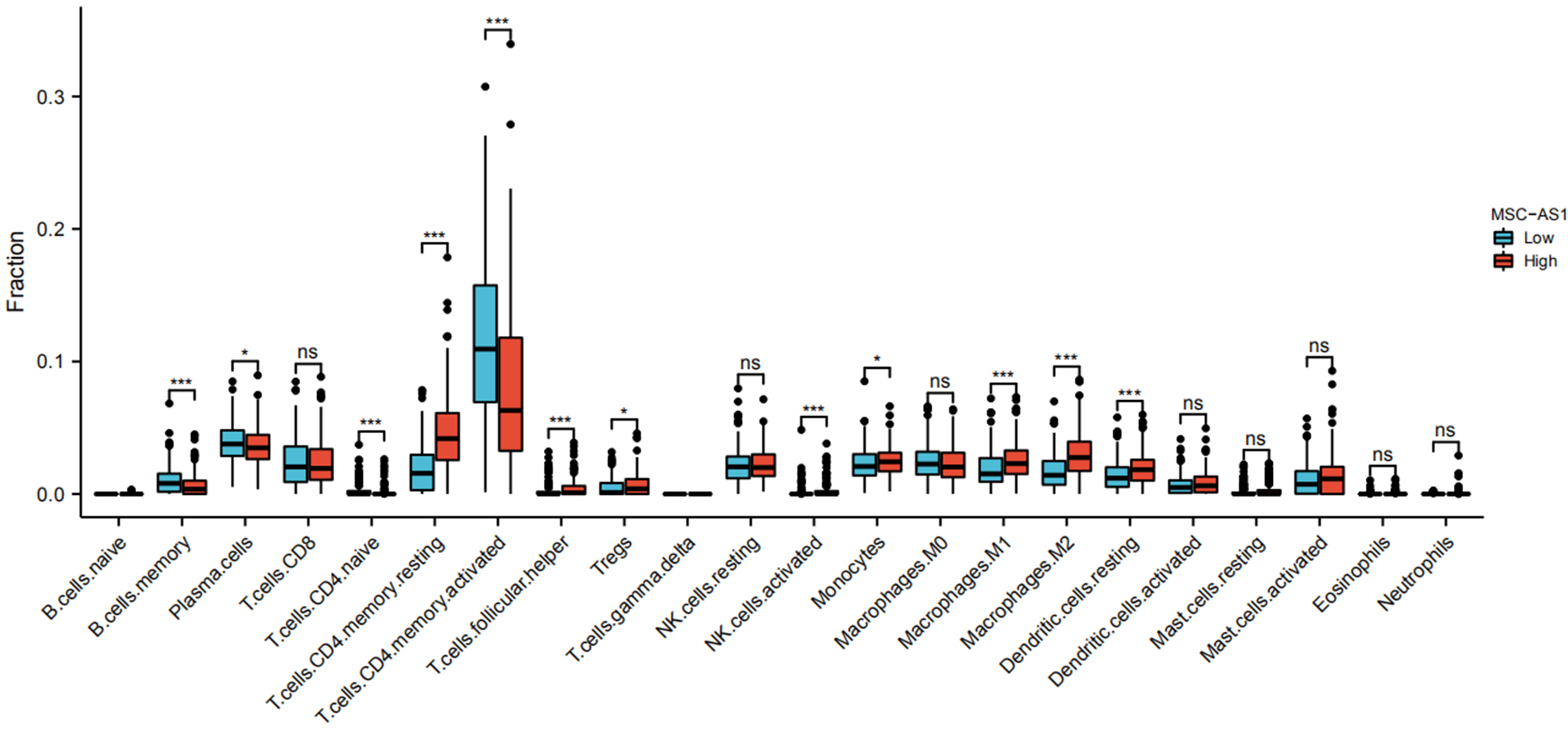

Supplement: Supplementary file 7 [file Image_1.PNG]
